# Diagnostic performance of coronary calcifications on CT to rule out acute coronary syndrome in the emergency department

**DOI:** 10.1186/s12873-024-01038-2

**Published:** 2024-07-12

**Authors:** Julie Paget Sentagne, Mickaël Ohana, François Severac, Pierrick Le Borgne, Erik-André Sauleau, Pascal Bilbault, Sabrina Kepka

**Affiliations:** 1 Emergency Department, Hôpitaux Universitaires de Strasbourg, 1 Place de L’hôpital, CHRU of Strasbourg, 67091 Strasbourg, France; 2 ICUBE UMR 7357 CNRS, Équipe IMAGeS, 300 Bd Sébastien Brant, 67400 Strasbourg, Illkirch-Graffenstaden France; 3 Radiology Department, Nouvel Hôpital Civil, Hôpitaux Universitaires de Strasbourg, 1 Place de L’hôpital, 67091 Strasbourg, France; 4 Groupe Méthodes en Recherche Clinique (GMRC), Hôpitaux Universitaires de Strasbourg, 1 Place de L’hôpital, 67091 Strasbourg, France; 5 UMR 1260, INSERM / Université de Strasbourg CRBS, 1 Rue Eugene Boeckel, 67000 Strasbourg, France

**Keywords:** Ultra low dose chest Computed Tomography, Coronary artery calcifications, Acute coronary syndrome, Emergency department, Chest pain

## Abstract

**Background:**

At present, the diagnosis of acute coronary syndrome (ACS) can be made by emergency physicians using the usual complementary tests, since the current troponin and electrocardiogram (ECG) protocols have been extensively tested for their safety. However, the detection of coronary calcifications on CT associated with coronary obstruction may be of interest for the diagnostic strategy in the emergency department (ED). The aim of this study was to evaluate a strategy combining a non-ischemic ECG with an initial normal troponin assay and the diagnostic accuracy of chest CT in detecting coronary calcifications to rule out the presence of an acute coronary event in patients presenting with chest pain in the ED.

**Methods:**

This was a retrospective, single-center study carried out in an ED in France and included all patients over 18 years of age presenting with chest pain between 1 June 2021 and 31 December 2021 with a non-ischemic ECG and a negative first troponin assay. The primary endpoint was the diagnostic performance of the combing strategy in ruling out ACS. The secondary endpoints were the sensitivity and specificity of calcifications in acute coronary syndrome, comparison with the diagnostic performance of a second troponin assay and the rate of reconsultation, rehospitalisation and investigations within 2 months of the ED.

**Results:**

Of the 280 patients included, 141 didn’t have calcifications. A total of 14 events were found with a negative predictive value for the combining strategy of 99.8% [95%CI: 98.2 – 100]. Sensitivity and specificity were 98.4% [95%CI: 83.8 – 100] and 53% [95%CI: 47 – 58.9], respectively. Among patients with no calcification, 8.2% were admitted to hospital and none suffered an acute coronary event. A total of 36 patients (12.8%) consulted a doctor within 2 months, with 23 investigations, all of which were negative in the non-calcification group.

**Conclusions:**

A strategy combining the detection of coronary calcifications on chest CT in patients with a non-ischemic ECG and a single troponin assay is effective to rule out ACS in the ED, and may perform better then ECG and troponin alone.

## Background

For several years now, activity in emergency departments (EDs) has been rising steadily, with an average increase of 3% per year, posing a real problem of overcrowding calling into question the quality and safety of care [1, 2]. The meta-analysis by Morley et al. published in 2018 highlighted the multiple repercussions of ED overcrowding, increasing treatments delay, length of stay, mortality, errors which require development of effective diagnostic tools to simplify management algorithms [3].

Among all ED visits, 5% of patients consult for chest pain, making it a frequent reason for referral [4]. Moreover, diagnosis is complex as this symptom can be a sign of various pathologies, with a wide range of severity, from life-threatening chest pain due to aortic dissection or myocardial infarction (10% of chest pain in EDs) to benign anxiety attack [5]. One in ten untreated acute coronary events results in death within an hour [6]. This serious and fatal pathology must therefore be eliminated as a priority in the management strategy for chest pain. With this in mind, current studies are developing risk stratification tools. At present, ED management consists of an electrocardiogram (ECG) and a cardiac enzyme (troponin) assay. There is an abundant literature on the safety of this current protocol for diagnosing acute coronary syndrome (ACS) in accordance with the recommendations of the European Society of Cardiology [7].

In addition, coronary calcifications are an established cardiovascular risk factor, with numerous studies having shown a significant correlation between their presence and associated coronary obstruction [8–10]. These can be assessed by different calcium scores on chest CT, using visual or quantitative methods. A recent study demonstrated a 99.6% negative predictive value of the absence of coronary calcifications on chest CT to rule out obstructive coronary pathology in a population of patients presenting to the ED with chest pain [11]. The development of ultra-low-dose computed tomography (CT) now makes it possible to perform precise imaging rapidly while limiting exposure to radiation.

However, a strategy combining a chest CT scan to exclude coronary calcifications, a non-ischemic ECG and a single troponin assay has not been evaluated to exclude ACS without performing a second troponin assay, although it is recommended in some cases. We therefore evaluated the diagnostic performance of chest CT in detecting coronary calcifications in combination with a non-ischemic ECG and a negative first troponin assay to rule out the ACS under real-life condition in patients presenting with chest pain in the ED.

## Methods

We conducted a monocentric retrospective observational study in the University Hospital of Strasbourg, including consecutive adult patients admitted to the ED between June 01, 2021 and December 31, 2021 for typical chest pain or atypical symptoms compatible with a cardiac origin. These patients were required to have a normal initial evaluation with a non-ischemic electrocardiogram and a negative first troponin assay, as well as to have undergone a chest CT scan to complete chest pain assessment. These criteria define patients at low or intermediate risk of an acute coronary event according to the Baptist Health Chest Pain Protocol used in the Grandhi et al. study on which this research was based [11, 12]. Patients with ST-segment elevation myocardial infarction (STEMI) and non-ST-segment elevation myocardial infarction (NSTEMI) were considered high-risk [11, 12].

The primary endpoint was the negative predictive value of coronary calcifications detected on chest CT in patients presenting with chest pain at low or intermediate risk of an acute coronary event with normal electrocardiogram and a negative first troponin assay. The reference gold standard was the discharge diagnosis attesting or not to an acute coronary event. This diagnosis was established by physicians at hospital discharge based on a combination of clinical, biological findings (second positive troponin) or coronary lesions highlighted in the different investigations. The secondary endpoints were:

- sensitivity and specificity of the detection of calcifications on chest CT for the diagnosis of acute coronary syndrome
- the relationship between the diagnostic performance indices of a second troponin at 3-h assay in the ED for the detection of acute coronary events in patients from the study population and those of calcifications
- the rate of re-consultations or rehospitalizations for cardiac reasons within two months, and the rate of cardiac investigations with evidence of coronary lesions within two months.

Data were collected directly from patient records extracted from electronic charts. Typical chest pain was described as retrosternal, oppressive and radiating into the left upper limb as defined by the European Society of Cardiology. Atypical symptoms included epigastric chest pain, right or bilateral laterothoracic pain, faintness, or digestive signs associated. The electrocardiogram was considered unchanged if there was no acute abnormality suggestive of an acute coronary syndrome (ST-segment elevation or depression, unknown conduction disorder bundle branch block or intraventricular conduction delay or repolarization disorder), or if there was no change in pre-existing abnormalities identified on prior ECGs [7].

Chest CT scans were retrospectively read by an emergency physician and a chest radiologist. The presence of coronary calcification was assessed in a binary manner (presence or absence) and then quantified using the visual "ordinal score", which gives a score of 0 to 3 for all 4 coronary arteries (left main, left anterior descending, circumflex and right coronary artery) and a final score ranging from 0 to 12 [13–15].

To assess the diagnostic performance of CT, the study evaluated the negative predictive value of coronary calcifications detected on chest CT in patients presenting with chest pain to EDs with absence of ECG changes and first troponin not increased.

A simulation based on the expected precision of the negative predictive value (NPV) was estimated. Based on the Grandhi et al. study [11] which found a negative predictive value (NPV) of 99% and assuming that 95% of subjects would present a negative test (no evidence of coronary calcifications on CT), the inclusion of 280 subjects would enable to estimate the NPV with a precision (size of the 95% credibility intervals) of 2.3% [95% CI: 0.7 – 3.6]. Continuous variables are presented as medians with interquartile ranges. Categorical variables are described using counts and percentages. Bayesian methods were used to assess the performance of the chest CT for the diagnosis of an acute coronary event. We used a Dirichlet distribution to estimate the cell probabilities from the two-way contingency table and to compute the negative predictive value and the other performance indicators (sensitivity and specificity). The estimation of the performance was performed using a vague prior corresponding to the Jeffreys prior (Dirichlet with α_i_ = ½, i = 1,…,4). Results are presented as point estimates with their 95% credibility intervals. All the analyses were performed using the R software.

The study was based on the recommendations of the European Society of Cardiology [7]. It was approved by local Ethics Committee (CE N°2022–99). In accordance with French low, no written consent was requested given the retrospective nature of the study.

## Results

**Fig. 1:**
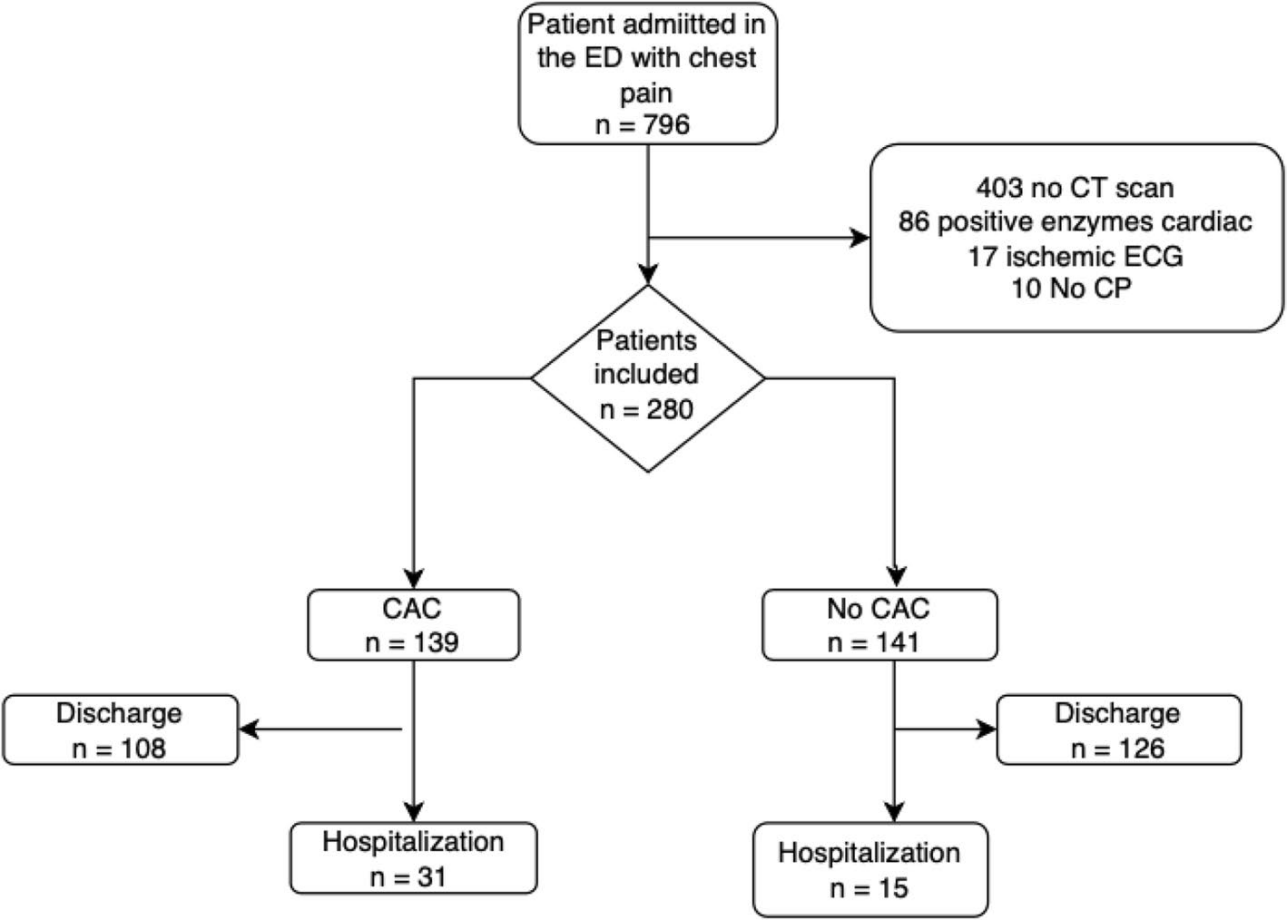
Flowchart of the study population. CAC: Coronary Artery Calcification; CP: Chest Pain; ECG: Electrocardiogram; ED: Emergency Departement

During the period of inclusion, 796 patients were admitted in the ED for chest pain. A total of 280 patient (130 women (46.4%), 150 men (53.6%), median age 61.5 years) had a non-modified electrocardiogram with a first negative troponin dosage and a chest CT performed in the ED (Fig. 1). Among the 280 patients, coronary artery calcifications were found in 139 patients (50%) and the average ordinal score to quantify calcifications was 7.8 [95%CI: 3.9 – 11.7].

The baseline characteristics of the study population are shown in Table 1. Among all patients, 154 described a typical chest pain (55%). Regarding cardiovascular risk factors, one on three patients had 3 risk factors or more. No ECG abnormalities were found in 72.9% of patients, and some known changes such as bundle branch block or negative T waves were found in the remainder.

**Table 1:** Characteristics of the study population

Characteristics		**Overall population** ***N* = 280**	**Group with calcifications** ***N* = 139**	**Group without calcification** ***N* = 141**
Age median, [quartiles]		61.5, [46.0, 74.25]	71.0 [61.0, 80.0]	48.0 [34.0, 62.0]
Gender, n (%)	Men	150 (53.6)	87 (62.6)	63 (44.7)
	Women	130 (46.4)	52 (37.4)	78 (55.3)
Typical CP, n (%)		154 (55)	86 (61 .9)	68 (48.2)
**Cardiovascular risk**				
Smoking, n (%)		119 (42.5)	59 (42.4)	60 (42.6)
Diabetes, n (%)		67 (23.9)	54 (38.8)	13 (9.2)
High Blood Pressure, n (%)		132 (47.1)	94 (67.6	38 (27.0)
Dyslipidemia, n (%)		113 (40.3)	81 (58.7)	32 (22.7)
Family history, n (%)		24 (8.6)	13 (9.4)	11 (7.8)
Obesity (IMC > 30), n (%)		76 (27.1)	39 (28.0)	37 (26.2)
**Paraclinical exam**				
Second troponin negative, n (%)		273 (97.5)	133 (95.7)	140 (99.2)
Normal ECG, n (%)		204 (72.9)	78 (56.1)	126 (89.4)
Ultralow dose scan, n (%)		168 (60)	89 (64.0)	79 (56.0)
Chest CT scan, n (%)		95 (33.9)	40 (28.8)	55 (39.0)
CT pulmonary angiography, n (%)		17 (6.1)	10 (7.2)	7 (5.0)
**Outcomes**				
Calcifications, n (%)		139 (49.6)		
Hospitalization, n (%)		46 (16.4)	31 (22.3)	15 (10.6)
Consult for cardiac issue (2 months), n (%)		36 (12.9)	25 (18.0)	11 (7.8)
Investigation (2 months), n (%)		23 (8.2)	19 (13.2)	4 (2.8)

CT scans were mainly ultra-low dose thoracic scan (60%) There were also scans with contrast product injection, including CT pulmonary angiography in which the injection protocol is adapted to specially visualize the pulmonary vessels and rule out pulmonary embolism.

There was a total of 14 (9,1%) diagnoses of acute coronary syndrome or equivalent, all among the patient with coronary calcifications.

The negative predictive value of the strategy combining coronary calcifications detected on chest CT, a non-ischemic ECG and a negative first troponin assay in this cohort was 99.8% [95%CI: 98.2 – 100], the gold standard being the discharge diagnosis attesting or not to an acute coronary event.

For the secondary endpoints, the specificity and the sensitivity of coronary calcifications detected on chest CT for the diagnosis of acute coronary syndrome were respectively 53% [95%CI: 47 – 58.9], and 98.4% [95%CI: 83.8 – 100].

About the diagnostic performance of a second troponin assay among the patients with coronary calcifications for the detection of acute coronary events, 273 patients (97.5%) had a second troponin negative and the negative predictive value of troponins calculated was 96.0%.

**Fig. 2:**
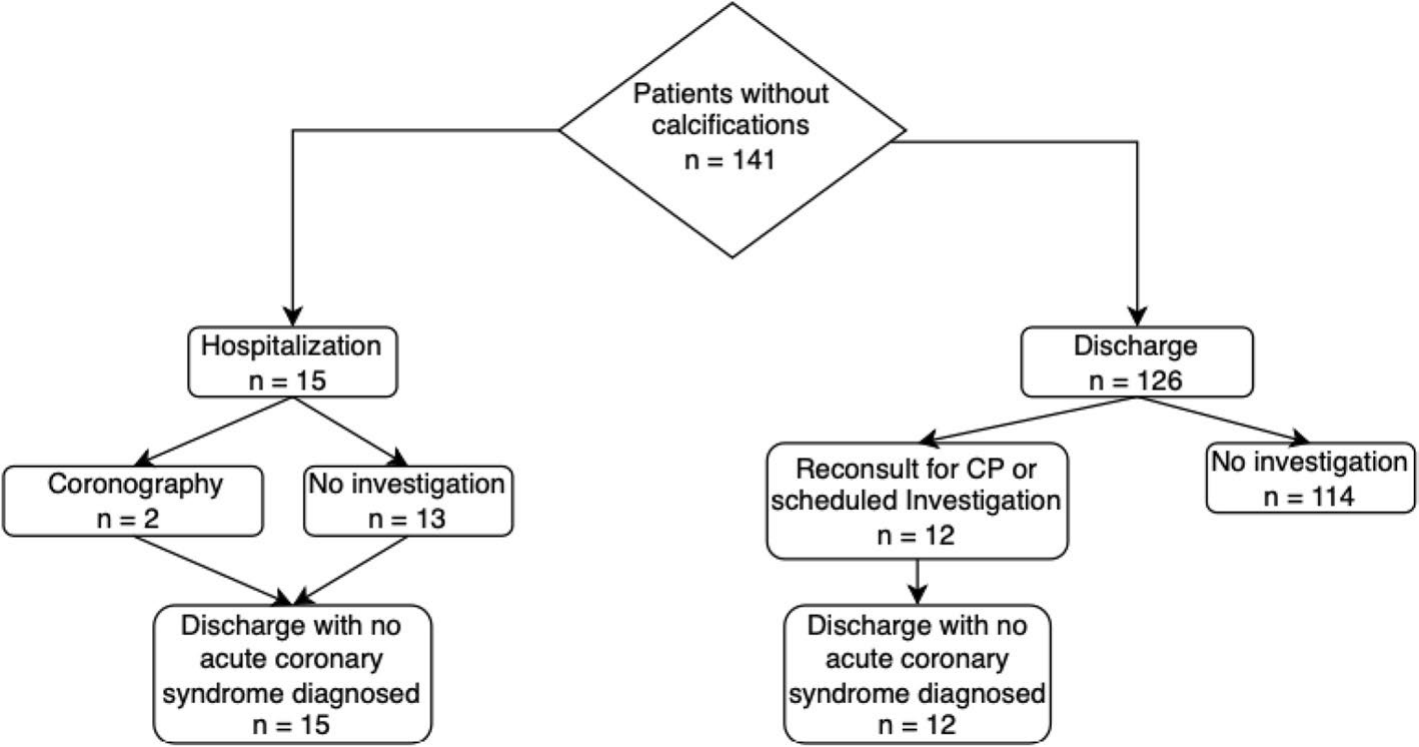
Healthcare pathway for patient without calcifications. CP: Chest Pain; Positive investigation: lesions which implied angioplasty or modification treatment

Finally, in the group of patients without calcification, 15 were hospitalized (10 in cardiology unit) including 2 who underwent invasive angio-coronarography (ICA). The 15 patients were finally discharge with no acute coronary syndrome diagnosed (Fig. 2).

**Fig. 3:**
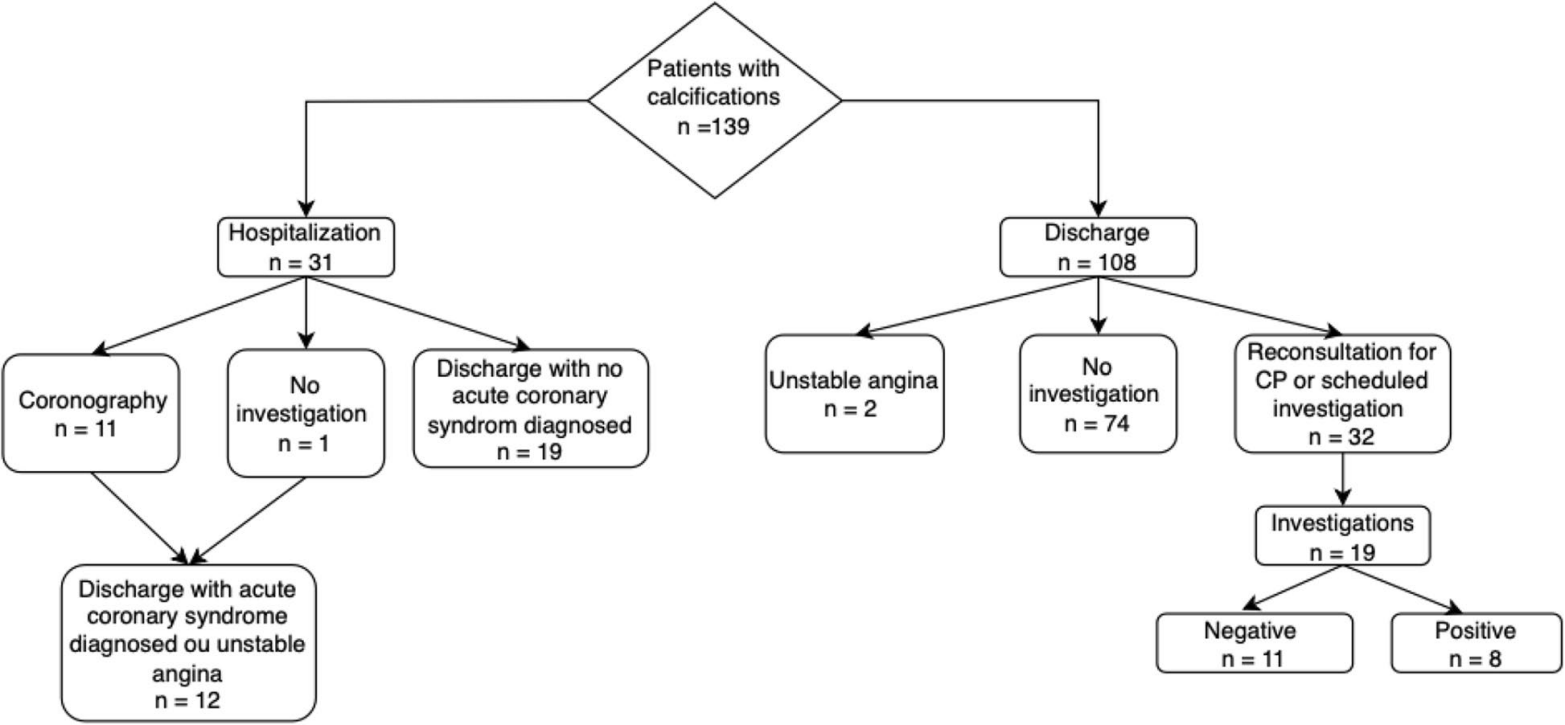
Healthcare pathway for patient with calcifications. CP: Chest Pain; Positive investigation: unknown coronary stenosis highlighted which implied angioplasty or modification treatment

One in five patients with calcifications on their CT scans required hospitalization, 75% of them in cardiology unit. Among the 31 inpatients, 11 underwent ICA, 8 needed an angioplasty and 3 were medically treated (Fig. 3). Furthermore, among the patients who underwent investigations during the two months following the passage in the ED (*n* = 23), 4 of them were included in the group without calcification and were negative so, no coronary stenosis was highlighted. On the other hand, 19 investigations were scheduled in the group with calcification and 8 of them presented some lesions which required percutaneous intervention (PCI) in 3 and modifications of their usual treatment in 5.

## Discussion

This study revealed that a strategy combining a negative first troponin assay, a non-ischemic ECG and a chest CT in ED for detecting coronary calcifications seemed effective to rule out an acute coronary event, in real-life conditions for patients presenting with chest pain. The negative predictive value of the combining strategy was 99.8%, considering the discharge diagnosis as the gold standard. Furthermore, among the inpatients without calcifications, there was no diagnosis of acute coronary syndrome (2 patients underwent ICA, with no abnormalities). Finally, of the 126 patients discharged home from the ED without calcifications, 10 patients re-consulted for chest pain and 2 had scheduled investigations and there was no diagnosis of acute coronary syndrome.

Thus, the results of this study confirm the high negative predictive value of coronary calcifications in ruling out acute coronary syndrome [11]. Indeed, compared with study by Grandhi et al., sensitivity and specificity are similar (98% versus 96% and 53% versus 62% respectively) [11]. The study by Litt et al., based on the diagnostic performance of calcifications on coroscans, also found a high negative predictive value of 100% to rule out coronary pathology in low-risk patients [16].

In the light of these findings, coronary calcifications could be a tool in a combining strategy for stratification of acute coronary events in ED, even though only a few studies exist in this context of acute chest pain. The main previous studies on thoracic imaging to rule out coronary obstruction have focused on the coronary CTA, particularly in the USA. It has repeatedly demonstrated its efficacy, particularly in reducing both length of stay and hospitalization rates [17, 18]. Until now, research on calcifications has focused on assessing overall cardiovascular risk. In fact, the Rotterdam study in 2012 confirmed the use of coronary calcifications to stratify patients' cardiovascular risk over the long term [19]. Recently, the study of Cury et al. highlighted the safety of discharging a patient with chest pain and a calcification-free coroscan [12]. However, the main limitation of this imaging modality remains irradiation, availibility and the need to inject of contrast product, a limiting factor for a significant number of patients in ED. Another way of using coronary calcifications for triage in the ED is to use a regular or a low-dose chest CT. Ultra-low dose (ULD) CT have the advantage of being up to 10 times less irradiating than the regular chest CT [20], and only slightly more irradiating than chest X-rays, while allowing a much more precise analysis of the lung parenchyma than the latter. It not only detects calcifications efficiently, but also eliminates differential diagnoses such as pneumothorax, pleural effusion, pneumopathy or bone involvement [21, 22]. The use of ULD CT is probably a solution for improving patient care in the ED, with less radiation as was the case in our study population who benefited of ULD in 60%.

Moreover, the prevalence of calcifications in the study population was higher than expected. Previous studies have found a lower prevalence than in our cohort [11, 23, 24]. The systematic review of Shreya et al. which studied the link between coronary calcifications and coronary pathology highlighted prevalence rates of calcification between 20 and 49%. This difference can be explained by the characteristics of the population studied. Indeed, 1 in 3 patients had at least 3 cardiovascular risk factors. Coronary calcifications are more frequent in high-risk vascular patients [25]. Furthermore, average age was also higher in our study than in the study of Grandhi et al. (61.5 versus 53 years). Similarly, several studies have shown an increase in calcifications with age [25, 26].

Finally, only 14 events were found in this study, which was expected given the target population. However, the inclusion of a sufficient number of patients enabled a good estimate to be made of the negative predictive value of calcifications.

### Limitations

First, half of the patients could not be included in this retrospective study due to the absence of CT scans. Despite this may limit the extrapolation of results, we still achieved a good accuracy of estimation in a large population. More, due to the retrospective nature of this study, some data could not be collected, as the time taken to obtain a CT which could have been relevant, in order to accurately assess the organizational consequences.

In addition, the occurrence of MACE one month after hospital discharge is usually considered the endpoint in previous studies. However, the design of the study meant that certain patient follow-up information was not collected, making it impossible to take this outcome into account in this retrospective study. Indeed, in our paper, the diagnosis of ACS was based on the discharge diagnosis, but it should be borne in mind that this may be influenced by the CT scan result. However, the aim of our study was to evaluate the diagnostic performance of CT calcification detection under real-life conditions, with a pragmatic objective: to assess the contribution of CT to the diagnostic strategy in the ED, which could enable ACS to be excluded without a second troponin assay, which is recommended in some cases.

Furthermore, the cardiovascular risk stratification of the study population was based on the American protocol of the Grandhi study. The use of a validated and recognized score such as the TIMI score would have been more relevant [27]. In fact, the TIMI risk score uses clinical data (age, coronary artery disease risk factors, severe angina symptoms, ST-deviation, elevated cardiac enzymes, and use of aspirin in the last seven days) to predict the short-term risk of acute myocardial infarction or coronary revascularization but some elements could not be collected in this retrospective study to calculate the TIMI score. In addition, the aim of this study was to evaluate a real-life management algorithm that was in line with current practice and easy to use. Finally, the emergency physician must maintain a clinical awareness and remain alert to the absence of calcification in certain coronary disease such as spontaneous coronary dissection and coronary embolism [28].

Despite these limitations, this study suggested the possibility of using calcifications on chest CT to rule out acute coronary syndrome. It highlights a stratification tool in real-life condition that is directly accessible to the emergency physician and reproducible, which is interesting in terms of rapid management. However, organizational benefits must be explored with the implementation in routine use of ULD chest CT. If the emergency exit time is shortened after the result of the ULD chest CT without a second troponin assay, this could be an argument in favor of the widespread use of ULD scanners in EDs for this indication, as it would enhance diagnostic performance while offering organizational advantages. Furthermore, ED overcrowding is associated with an increase of hospital LOS and mortality [29, 30]. Implementation of measures to reduce ED stay is a way to improve outcomes for patients admitted in the ED. This implies that priority must be given to shorten access times for imaging by specific healthcare pathways between ED and radiology.

## Conclusion

The encouraging results of this real-life condition study suggests that the assessment of coronary calcifications on CT combined with a single troponin assay in patients with a non-ischemic ECG and negative first troponin assay appears to be effective for excluding acute coronary syndrome. Although larger prospective studies are necessary to confirm these results, this strategy is of real interest in the ED for patient triage, particularly in the current context of overcrowding, and should be part of the pathway dedicated to the management of chest pain.

## Data Availability

All data generated or analysed during this study are included in this published article.

## Abbreviations

EDs: Emergency Departments
ECG: Electrocardiogram
CT: Computed Tomography
ULD: Ultra-Low-Dose
NPV: Negative Predictive Value
CAC: Coronary Artery Calcification
CP: Chest Pain
